# Supplementary material for: Stability of Human Telomeric G-Quadruplexes Complexed with Photosensitive Ligands and Irradiated with Visible Light
Source: Int J Mol Sci. 2023 May 22;24(10):9090. doi: 10.3390/ijms24109090 (PMC10218862; doi:10.3390/ijms24109090)
Supplement: Supplementary file 1 [file ijms-24-09090-s001.zip › ijms-2394457-supplementary.pdf]

International Journal of *Molecular Sciences*

MDPI

Supporting Material for "Stability of Human Telomeric G-Quadruplexes complexed with Photosensitive Ligands and Irradiated with Visible Light"

Valeria Libera [1,2,†], Francesca Ripanti [1,†], Caterina Petrillo [1], Francesco Sacchetti [1], Javier Ramos-Soriano [3], Maria Carmen Galan [3], Giorgio Schirò [4], Alessandro Paciaroni [1,*], and Lucia Comez [2,*]

1 Department of Physics and Geology, University of Perugia, Via Alessandro Pascoli, 06123 Perugia, Italy; valeria.libera@studenti.unipg.it (V.L.)
2 Italian National Research Council-Istituto Officina dei Materiali(IOM) c/o Department of Physics and Geology, University of Perugia, , Via Alessandro Pascoli, 06123 Perugia, Italy
3 School of Chemistry, University of Bristol, Cantock's Close, Bristol BS8 1TS, UK
4 CNRS, CEA, IBS, c/o University Grenoble Alpes, 38400 Grenoble, France
* Correspondence: alessandro.paciaroni@unipg.it (A.P.); comez@iom.cnr.it (L.C.)
† These authors contributed equally to this work.

S1. Experimental setup

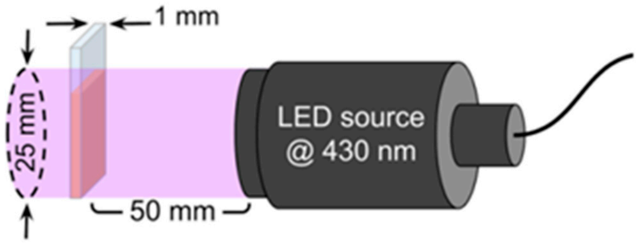

Figure S1. Custom-made setup for illumination. The entire setup is contained in a dark box and all the parts are fixed to high-precision stages to guarantee reproducibility and stability of the measurements.

S2. Reversible properties of DTE ligand

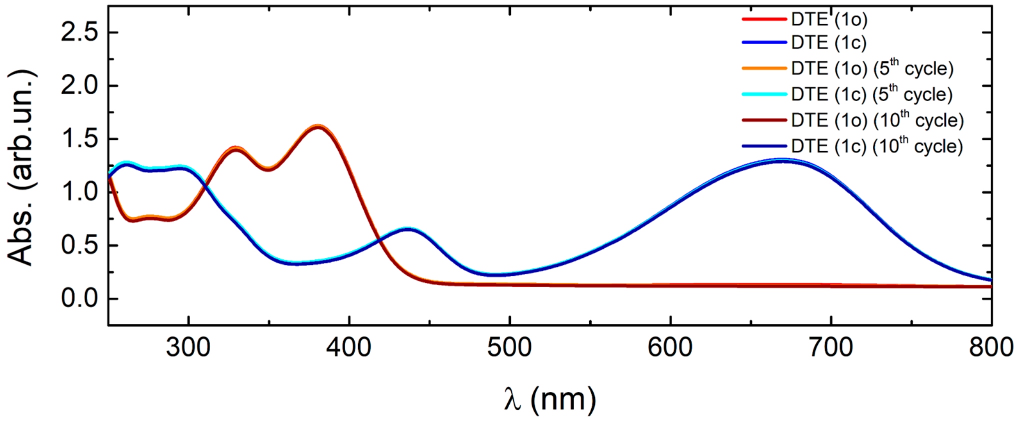

Figure S2. Absorption spectra of DTE (1o) and (1c) after different irradiation cycles. The process is perfectly reversible and reproducible for several cycles.

S3. Titration experiment on Tel22-DTE (1o) complex

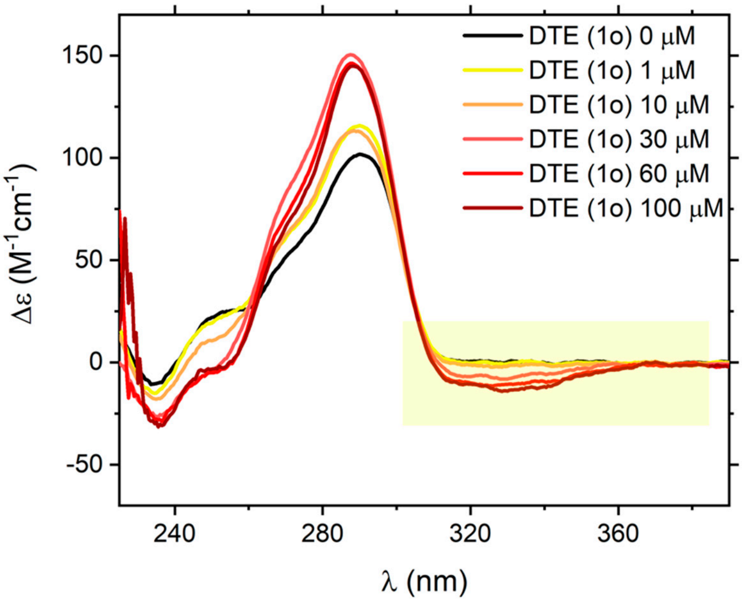

Figure S3. CD titration measurements of Tel22 at a fixed concentration of 30 μM and gradually increasing amount of DTE (1o).

S4. SAXS experiments

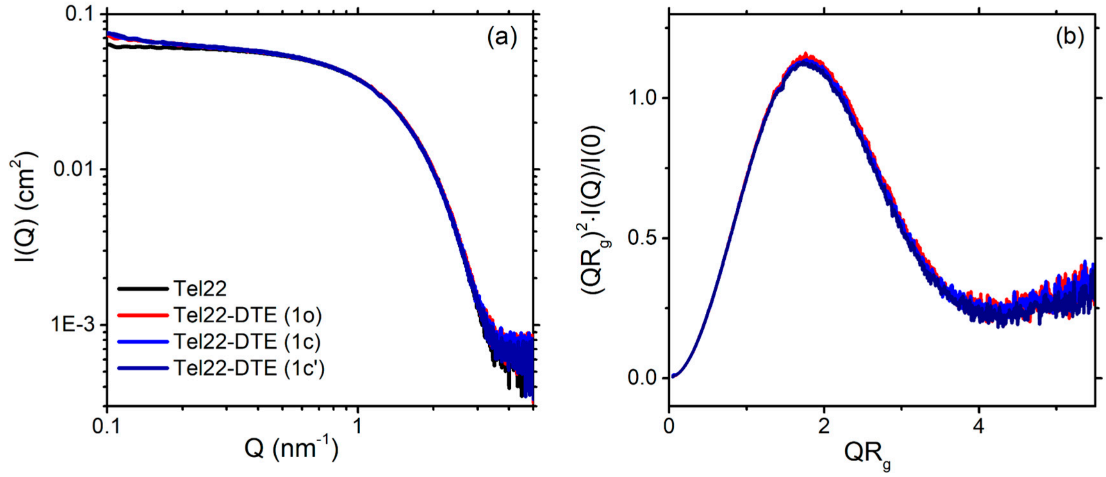

Figure S4. (a) SAXS patterns recorded at BM29 beamline (ESRF, https://www.esrf.fr/) of Tel22 and Tel22-DTE complexes. (b) Corresponding dimensionless Kratky plot. The peak position at 1.75 with the height of $3/e \approx 1.1$ indicates a compact, folded structure.

S5. Application of CD deconvolution algorithm to Tel22 and Tel22-ligand complexes

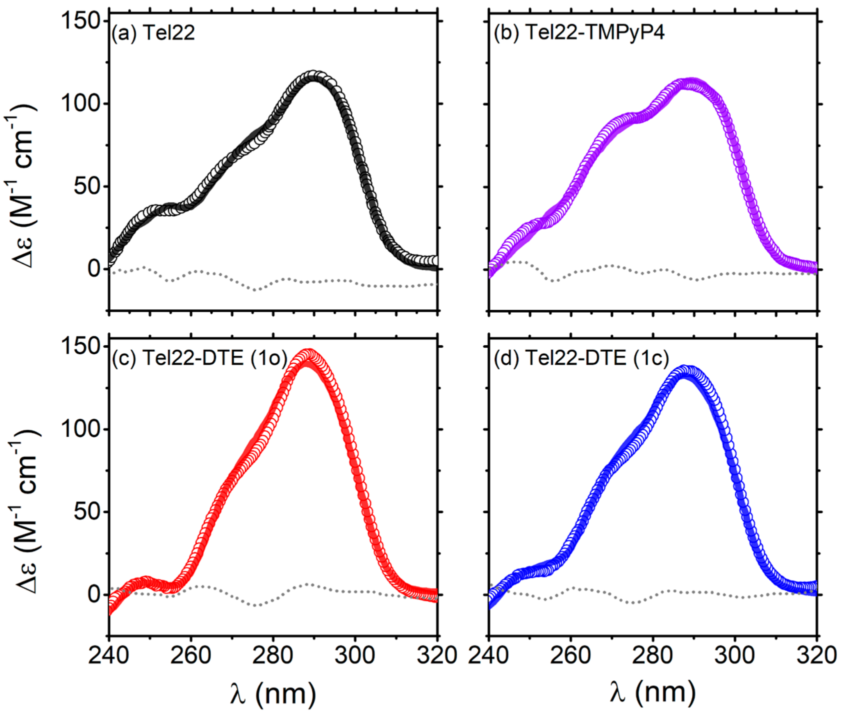

Figure S5. CD experimental (symbols) and theoretical (lines) fit curves obtained by using the routine developed in Ref. [1]. Residuals are represented as dots.

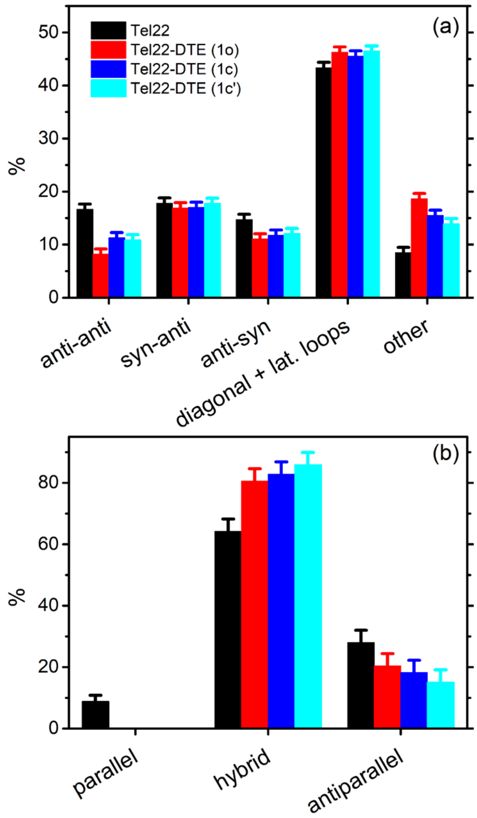

Figure S6. Percentage of components obtained from the analysis of CD spectra by means of the routine developed in Ref. [S1]: (a) secondary and (b) tertiary structure contributions.

S6. Blue light illumination of Tel22-TMPyP4 complex

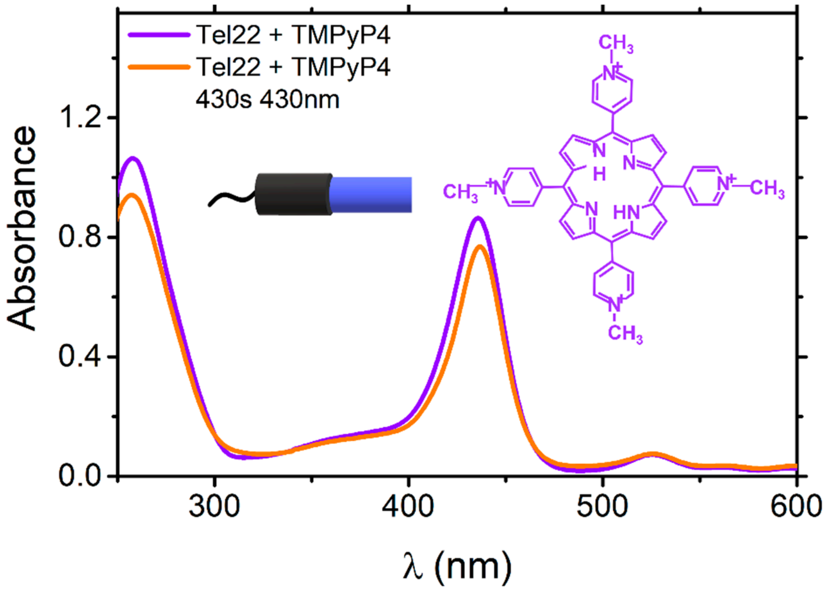

Figure S7. Absorption spectra of Tel22-TMPyP4 before (purple) and after (orange) 480 s irradiation with blue light.

S7. Interpretation of melting pathways

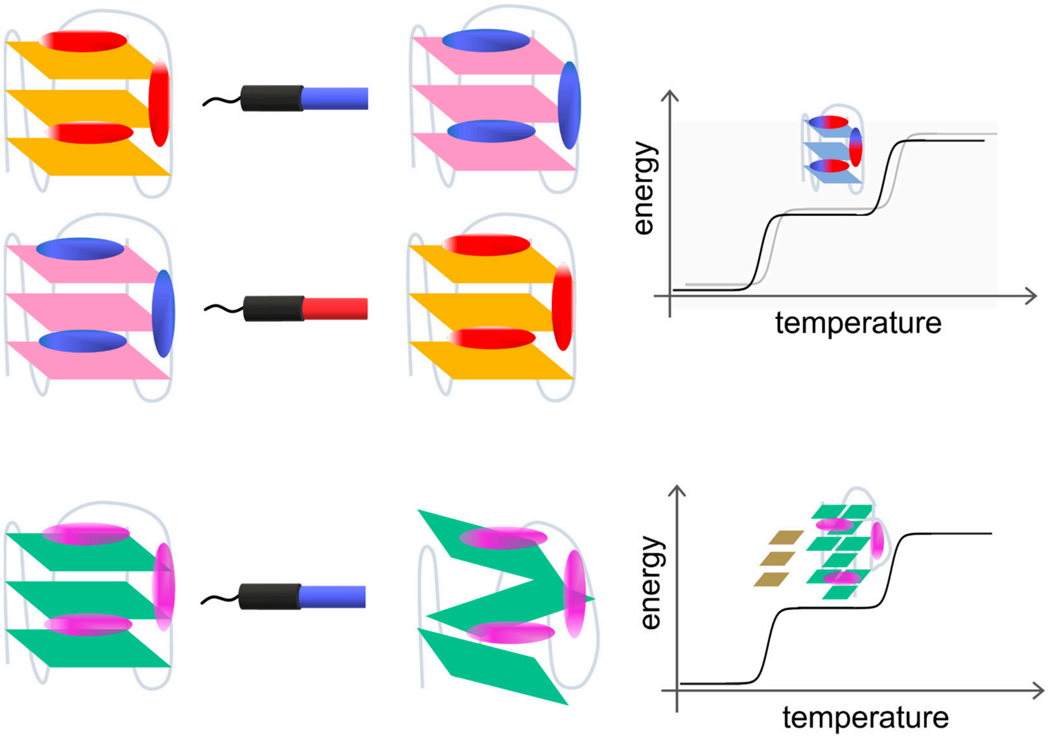

Figure S8. Schematic representation of the thermal pathway for Tel22-DTE (1o) and Tel22-DTE (1c) and for Tel22-TMPyP4 complexes.

	ΔH_1 (Kcal/mol)	ΔH_2 (Kcal/mol)	ΔH_3 (Kcal/mol)	T_{m1} (K)	T_{m2} (K)	T_{m3} (K)
Tel22-DTE (1o)	-37±3	-12.6±1.2	-50±4	313±2	341±2	348±2
Tel22-DTE (1c)	-15.9±1.3	-23±2	-50.9±4.1	317.6±2.1	340±2	349±2

Table S1. Thermodynamic parameters obtained from SVD analysis of Tel22-DTE samples.

	ΔH_1 (Kcal/mol)	ΔH_2 (Kcal/mol)	T_{m1} (K)	T_{m2} (K)
Tel22-TMPyP4	-26±2	-59±5	323±2	344.5±1.9
Tel22-TMPyP4 i.t. 30s 430 nm	-26±2	-51±4	321.5±1.8	345.5±1.8
Tel22-TMPyP4 i.t. 120s 430 nm	-23±2	-48±3	323±2	344±2
Tel22-TMPyP4 i.t. 480s 430 nm	-21±2	-42±3	323±2	339±2

Table S2. Thermodynamic parameters obtained from SVD analysis of Tel22-TMPyP4 samples.

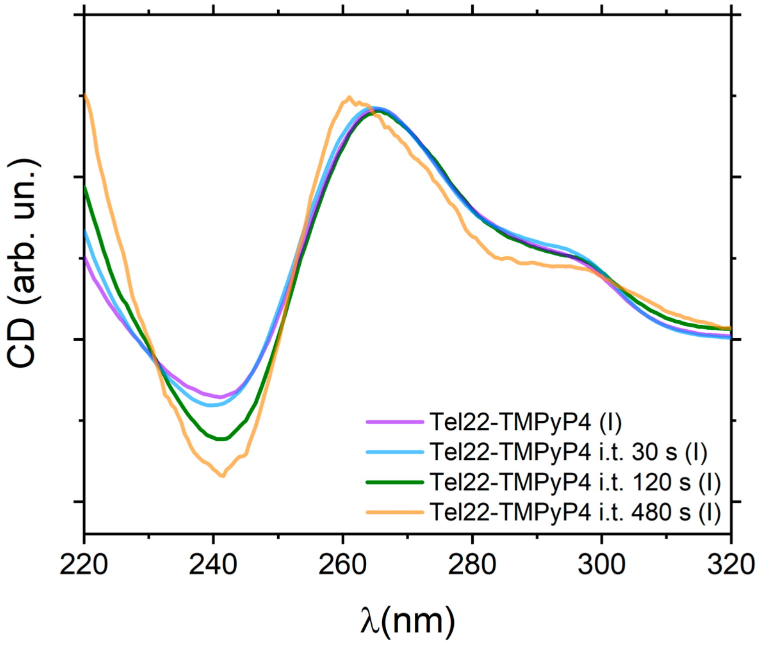

Figure S9. The same CD profiles of Figure 6 reproducing the different intermediate states, reconstructed via SVD, normalized for sake of comparison.

S8. Tel22-DTE in Na^+ environment

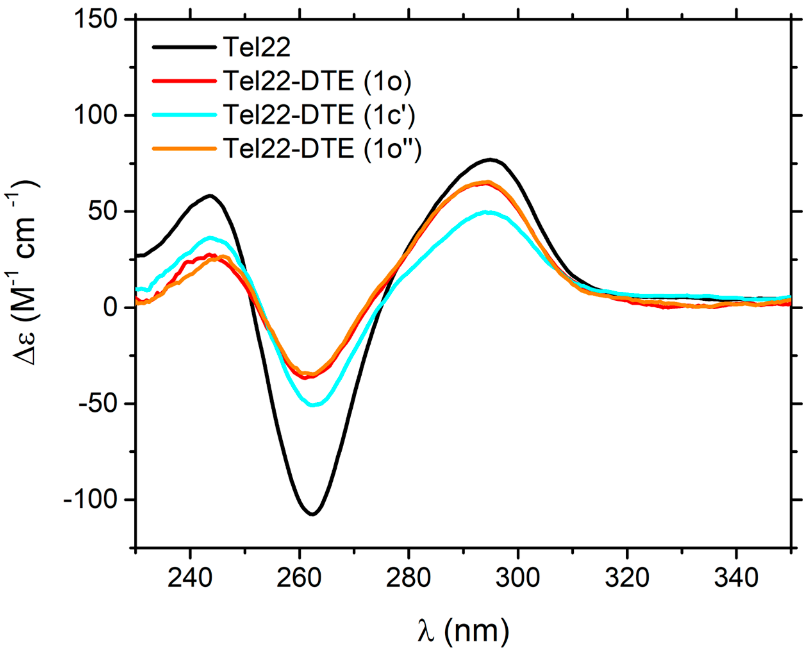

Figure S10. CD spectra of Tel22 (black), Tel22-DTE (1o) (red), and Tel22-DTE (1c′) (blue) obtained from Tel22-DTE (1o) after i.t. 10 min at 430 nm, and Tel22-DTE (1o′′) obtained from the previous one after i.t. 90 min at 660 nm. All the samples were prepared in Na^+ buffer. These measurements indicate the complete reversibility of Tel22-DTE complexes in this environment.

S9. Blue light illumination of Tel22-DTE complex

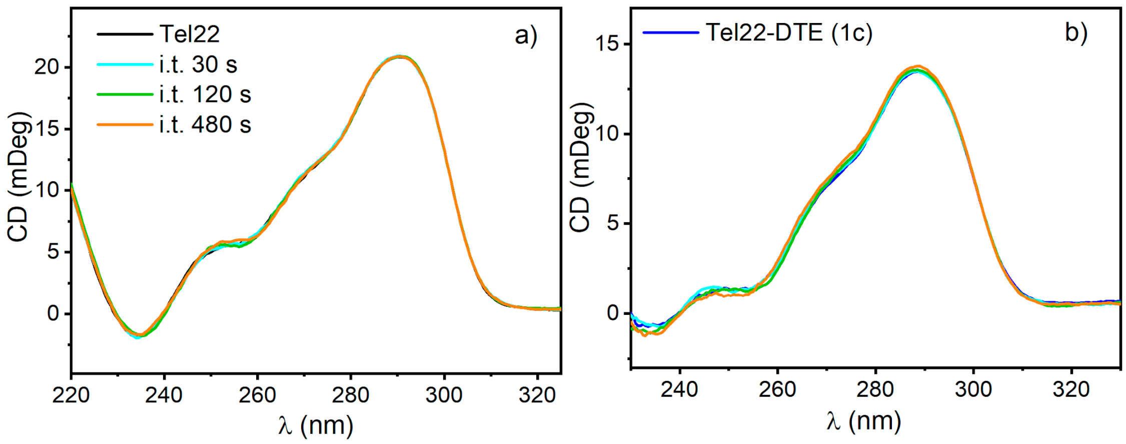

Figure S11. a) CD spectra of Tel22 (black), Tel22 i.t. 30 s (light blue), Tel22 i.t. 120 s (green), Tel22 i.t. 480 s (orange). b) CD spectra of Tel22-DTE (1c) (blue), Tel22-DTE (1c) i.t. 30 s (light blue), Tel22-DTE (1c) i.t. 120 s (green), Tel22-DTE (1c) i.t. 480 s (orange).

S10. SVD applied to Tel22-DTE (1o) melting spectra

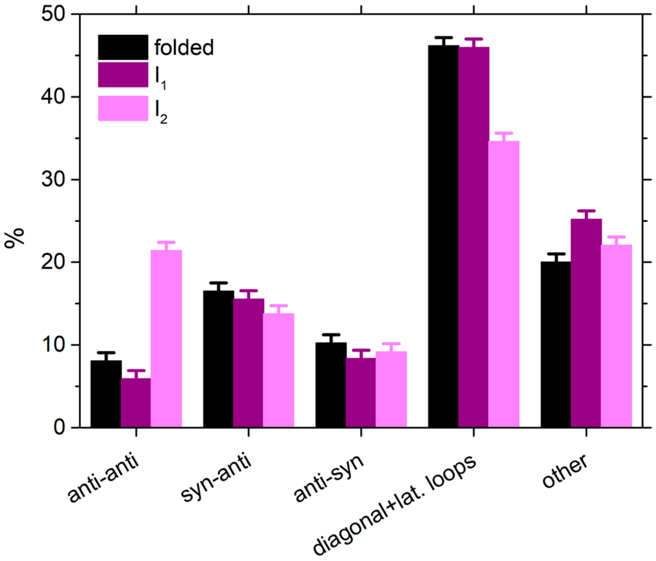

Figure S12. Percentage of components obtained from the analysis of CD spectra upon melting of Tel22-DTE (1o) complex through the routine developed in Ref. [S1].

.

References

S1. Del Villar-Guerra, R.; Trent, J.O.; Chaires, J.B. G-Quadruplex Secondary Structure Obtained from Circular Dichroism Spectroscopy. *Angew. Chem. Int. Ed Engl.* **2018**, *57*, 7171–7175.
